# Effect of Deep Placement Fertilization on Soybean (*Glycine max* L.) Development in Albic Black Soil

**DOI:** 10.3390/plants15030424

**Published:** 2026-01-30

**Authors:** Jiahe Zou, Qiuju Wang, Haibin Zhang, Qingying Meng, Jingyang Li, Aihui Chen, Xin Liu, Yifei Luo, Zhenhua Guo

**Affiliations:** 1 Heilongjiang Provincial Key Laboratory of Soil Environment and Plant Nutrition, Heilongjiang Academy of Agricultural Sciences, Harbin 150086, China; 2 Animal Husbandry Research Institute, Heilongjiang Academy of Agricultural Sciences, Harbin 150086, China

**Keywords:** abscisic acid, ground vegetation, metabolomics, soil microbiome, transcriptomics

## Abstract

Maximizing the agricultural output on inherently infertile land and minimizing the environmental cost remain central research imperatives. Albic black soil typifies such infertility. Conventional practice relies on fertilization and straw incorporation, but the albic layer’s impermeability funnels applied nutrients into adjacent aquatic systems. Therefore, this study developed deep placement fertilization by lodging fertilizer directly within the albic layer to block hydrologic loss. The feasibility of mechanization was first validated in pot experiments. Soybeans were allocated to six treatments simulating fertilizer placement at different soil depths: control (C), control and fertilizer (CF), surface soil mixing (SM), surface soil mixing and fertilizer (SMF), plow pan soil mixing (PM), and plow pan soil mixing and fertilizer (PMF). The treatments used 20 cm tillage, and the data were collected after 15, 25, and 35 days and at harvest. Integrative transcriptomic, proteomic, metabolomic, and soil microbiome profiling revealed that fertilizer positioned at 25 cm in the albic layer increased yield, restructured the rhizobiont community and promoted arbuscular mycorrhizal fungal colonization. Among the fertilizer treatments, CF had the best growth, and SMF was inhibited by a nutrient shortage. SMF and PMF lost water faster than CF. Abscisic acid (ABA) conveyed the subterranean fertilization signal to the leaf. The enrichment of *Vicinamibacterales*, *Xanthobacteraceae*, and *Glomeromycota* in soil lowered the ABA content in the roots, which upregulated thymidine kinase and peroxidase upon arrival in the leaf, increasing yield. These findings provide a transferable benchmark for any parent material exhibiting poor hydraulic conductivity.

## 1. Background

A latent cycle pervades the human-dominated biosphere: fertilizer over-application secures food [1], but the ensuing greenhouse flux warms the planet [2]; rising temperatures suppress photosynthesis and erode yield [3,4,5], prompting higher fertilizer doses and additional land conversion. Simultaneously, nutrient runoff triggers fluvial eutrophication [6,7]. Maintaining maximal crop output and minimizing the environmental cost has therefore become imperative for sustainable agriculture [1,8,9]. Therefore, viable pathways have emerged: continuous maize (*Zea mays* L.) cultivation that assembles a superior rhizobiont community [10] and rice systems whose phytohormonal gibberellin regime is precisely tuned [8].

**Figure 1:**
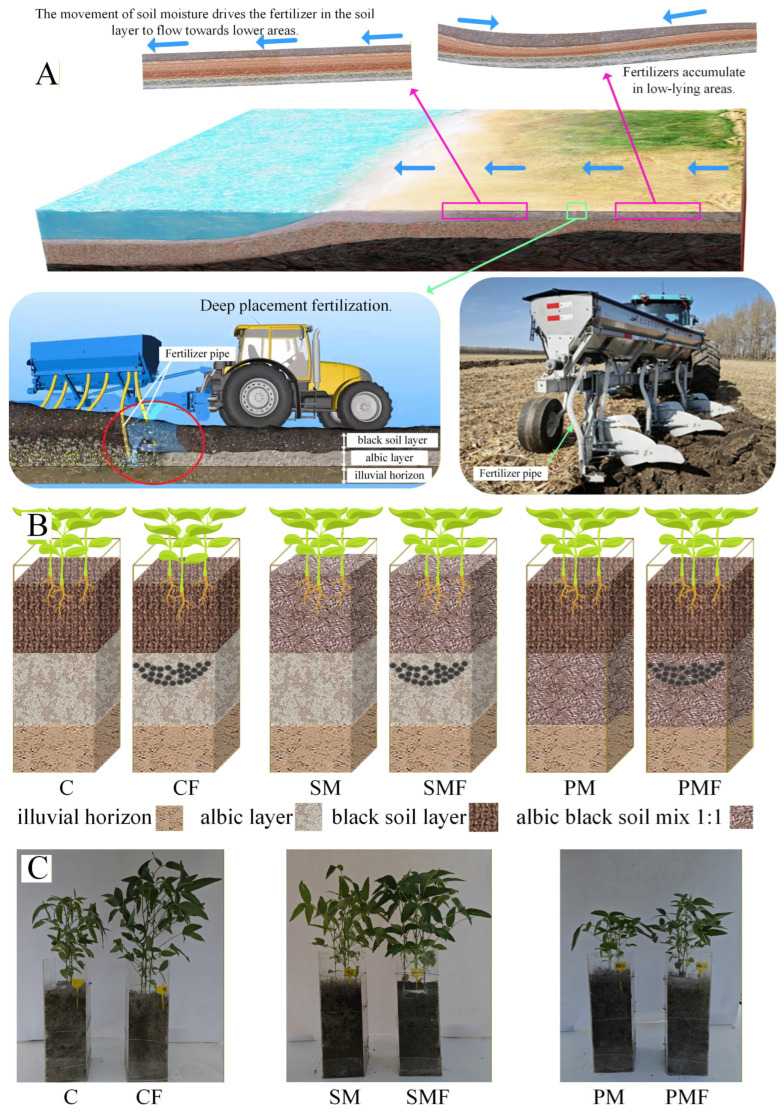
Experimental design and plant comparison photographs on day 35. (**A**) Surface fertilizer migration with soil water and the prototype deep release applicator (25 cm) developed in this study. (**B**) Schematic of treatment layout; black dots indicate fertilizer positioned at a 25 cm depth. (**C**) Photographs of all 6 groups on day 35; plexiglass box height = 60 cm.

Albic black soil (albic black Luvisols/argillic soil) covers 5–6 million ha in China and comparable areas worldwide [11,12,13,14,15]. Under native vegetation, it presents a three-tier profile: black topsoil, albic bleached horizon, and illuvial subsurface [16]. The albic horizon, which is low in nutrients and organic carbon, categorizes the soil as inherently low yield [11,13,14,15]. In cultivated fields, the plow layer is a mechanically admixed zone of black and albic materials. Its fertility remains marginal, and the agronomic consensus dictates minimal albic incorporation coupled with external nutrient input [12,13]. Amelioration research has progressed from deep plowing to refined fertilization strategies, such as straw retention, organic amendments, and biochar, in the past six decades [13]. Most studies have focused on surface fertilization because of the albic horizon’s shallow effective thickness, low water content, and poor permeability [13,14,17], spawning specialized application machinery [12,18]. The albic horizon’s extremely low hydraulic conductivity is the primary determinant of intrinsic infertility [11]; its poor drainage and aeration impose recurrent drought–waterlogging cycles that suppress yield [12]. Solutes deposited above this horizon are rapidly vectored into the hydrosphere via interflow (Figure 1A). Placement of slow-release boron at an 8 cm depth within the albic layer significantly increased the yield of *Beta vulgaris* L. [17], indicating that embedding nutrients inside the low-permeability zone can curtail off-site loss and sustain slow-release nutrition. Pot-scale corroboration has shown that albic/black admixtures amended with straw increased soybean (*Glycine max* L.) yield [12]. Soybean cultivation is very extensive in Heilongjiang Province, and many of the cultivated soils are black soil [19,20]; strictly speaking, it is the white slurry black soil mentioned in this study. Different fertilization methods can lead to differences in soybean growth [21], and multi-omics studies can reveal the physiological mechanisms of soybean growth differences [22].

Objective 1 of this study integrated multi-omics to assemble a dedicated rhizobiont community for soybean cultivation on albic soil. Continuous soybean monocropping reduces yield through progressive rhizosphere dysbiosis [23,24] and enriches the pathogenic genera *Thanatephorus* and *Fusarium*, precipitating disease outbreaks [25,26]. The conventional countermeasure is maize–soybean rotation [27,28,29], which increases maize yield by 13% and soybean yield by 25% compared to monoculture [27]. A previous study profiled the microbiome trajectory under such rotation [30]. Agriculture currently accounts for nearly one-fourth of global greenhouse gas emissions [4], and specific soil microbiota can attenuate these fluxes [2]. Therefore, it is necessary to generate mechanistic insight into the assembly and function of the soybean rhizobiont that simultaneously sustains yield and mitigates the environmental cost.

Objective 2 was to evaluate deep placement fertilization in albic soil to intercept surface-borne nutrient fluxes to fluvial systems. Figure 1A illustrates a prototype applicator that releases granulated fertilizer at operator-defined depths; however, the thickness of the overlying black topsoil is spatially heterogeneous [16], necessitating site-specific calibration of the injection depth. The resulting depth–response dataset will provide a mechanistic framework for precision sub-layer fertilization. Because albic black soil is classified as “Pseudo-gley” (Germany), “Lessivage” (France), or “pseudo-podzolic” (Russia) [12], the derived algorithms are directly transferable to any parent material exhibiting comparably impaired hydraulic conductivity.

## 2. Materials and Methods

### 2.1. Pot-Based Simulation of Stratified Soil Profiles

The experiment was conducted at Heilongjiang Provincial Key Laboratory of Soil Environment and Plant Nutrition, Harbin, China. Maize–soybean rotation is the dominant cropping system in northern China [30]; the soil used had grown maize in 2024. Eighteen rectangular plexiglass boxes (20 cm × 20 cm × 60 cm) were assigned to six treatments (Table 1, Figure 1B): control (C), control + fertilizer (CF), surface soil mixing (SM), surface soil mixing + fertilizer (SMF), plow pan mixing (PM), and plow pan mixing + fertilizer (PMF). Seeds of cultivar ‘Beihuidou 1#’ supplied by the Soybean Research Institute, Heilongjiang Academy of Agricultural Sciences (Harbin, China), were sown on 10 June 2025 (day 0) with 6 plants per box; an additional 50 plants were raised in spare pots. After 10 days, emergence was recorded, and each box was thinned to 3 or 4 uniform plants, with gaps refilled from the reserve. The plant height, stem thickness, and leaf chlorophyll content were recorded after 15, 25, and 35 days. The chlorophyll content was measured using a Youyunpu chlorophyll meter (Youyunpu Photoelectric Technology Co., Ltd. www.youyunpu.cn, Weifang, China) on six randomly selected leaves per treatment. After 35 days, leaves, root tissue, and rhizosphere soil were sampled from 4 randomly chosen plants per treatment for downstream analyses. The final harvest was performed on 20 September, and the number of grains, number of nodes, and yield per plant were recorded.

### 2.2. Deep Placement Fertilization Reconfigures Soybean Organ Architecture and Subcellular Ultrastructure

After 35 days, 4 leaf and root samples each from C and CF were subjected to histological sectioning and safranin–fast green double staining [31]. Briefly, tissues were fixed in medium (Servicebio Technology Co., Ltd. www.servicebio.cn, Wuhan, China) for 24 h, dehydrated, paraffin-embedded, sectioned, dewaxed, stained with safranin for 2 min, rinsed with distilled water, differentiated in graded ethanol, counterstained with fast green for 15 s, dehydrated in absolute ethanol, cleared in xylene, and mounted in neutral balsam.

Parallel tissues were processed for transmission electron microscopy (TEM) [32]. Samples were pre-fixed in 5% glutaraldehyde–4% paraformaldehyde for 24 h, post-fixed in 1% OsO_4_ (Osmium(VIII) oxide) for 4 h, dehydrated, resin-embedded, ultra-thin sectioned, and stained with uranyl acetate for 30 min and with lead citrate for 15 min. The subcellular architecture was examined at 80 kV.

### 2.3. Transcriptomic, Proteomic, and Metabolomic Analyses of Soybean Leaves

After 35 days, each group has 3 boxes (9 to 12 plants), and 4 leaves are collected from each box (3 to 4 plants). After mixing, a sample is made, with 3 samples in each group, and subjected to transcriptomic, proteomic, and metabolomic profiling. The following comparisons were performed: CF vs. C, SMF vs. SM, and PMF vs. PM.

#### 2.3.1. Sequencing Methods

Total RNA was extracted from leaf tissue with poly(A)-enriched with Oligo dT. Libraries were constructed and sequenced on the NovaSeq X Plus system (Illumina, Inc. San Diego, CA, USA).

For proteomics, leaf tissue was lysed to generate protein extracts; after quality control, peptides were prepared and analyzed by data-independent acquisition mass spectrometry on a Vanquish Neo UHPLC system (Thermo, Waltham, MA, USA) controlled by Xcalibur 4.7 (Thermo, Waltham, MA, USA).

For untargeted metabolomics, 50 mg leaf powder was lysed in 400 µL MeOH:H_2_O (4:1) at −10 °C and centrifuged (13,000× *g*, 15 min), and the supernatant was injected into a Q Exactive HF-X (Thermo, Waltham, MA, USA). Data were processed using Progenesis QI version 3.0 (La Jolla, CA, USA).

#### 2.3.2. Multi-Omics Integration

Volcano plots visualized differentially expressed genes (DEGs), differentially expressed proteins (DEPs), and differentially expressed metabolites (DEMs) under *p*-value < 0.05 with log_2_FC thresholds of ±1 (transcriptome), ±0.3 (proteome), and ±0.1 (metabolome). Up- and downregulated RNAs were subjected to Kyoto Encyclopedia of Genes and Genomes (KEGG) enrichment (*p*-value < 0.1), and the 10 most significant pathways per direction are reported.

Transcriptome– and proteome–phenotype associations: DEPs were filtered at fold-change <0.8 or >1.2 with *p*-value < 0.05. Transcripts whose log_2_FC direction matched the corresponding protein (significance not required) were retained. Protein–protein interaction (PPI) networks were constructed in STRING (https://string-db.org/, accessed on 7 September 2025), and Pearson correlations (|r| > 0.6, *p*-value < 0.05) between DEPs and phenotypic traits were calculated. Integrated visualization was performed in Cytoscape (version 3.7.2, web.cytoscape.org).

Proteome–metabolite associations: DEPs and DEMs were subjected to KEGG enrichment, and intersecting pathways were identified. Differential proteins and metabolites mapped to these shared pathways were extracted and correlated across samples.

### 2.4. Rhizosphere Microbiome Analysis

After 35 days, 4 rhizosphere soil samples (5 cm depth) were randomly collected from 6 groups for sequencing of the 16S rRNA and internal transcribed spacer (ITS) regions. CF vs. C, SMF vs. SM, and PMF vs. PM retained genera with >1% mean relative abundance, and genus-level heat maps were generated. Bacterial and fungal genera were independently correlated with yield and plotted as heat maps. Spearman correlations (|ρ| > 0.6, false discovery rate (FDR) < 0.05) among bacteria, fungi, and yield were calculated for the same taxa and visualized in Cytoscape.

### 2.5. Statistical Analysis

Soybean growth data were subjected to statistical analysis using the software SPSS (Statistical Package for the Social Sciences, version 31.0, IBM, New York, NY, USA). Fisher’s classic one-way analysis of variance (ANOVA) was applied to determine the significance of the data, *p*-value < 0.05 considered to have significant differences

## 3. Results

### 3.1. Effect of Deep Placement Fertilization on Soybean Development

**Figure 2:**
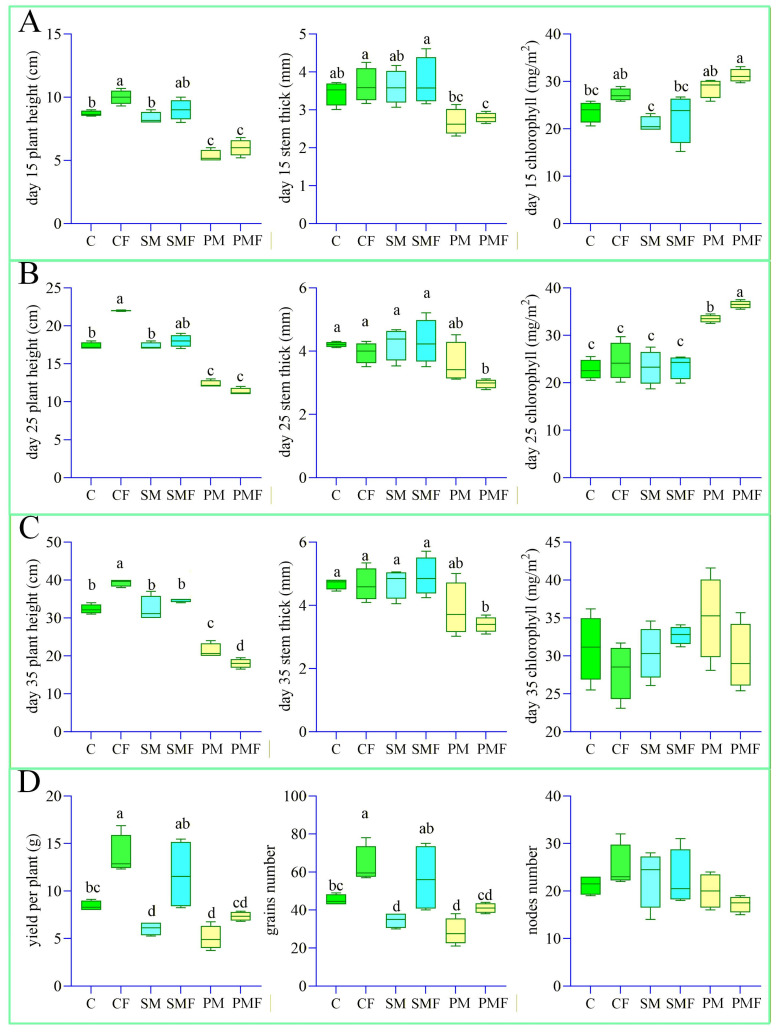
Box-and-whisker plots of soybean growth traits. Perform one-way analysis of variance (ANOVA) on soybean growth data from 6 groups. (**A**–**C**) Plant height, stem thickness, and leaf chlorophyll content from day 15 to 35. (**D**) Number of grains, number of nodes, and yield per plant at harvest. Different letters indicate significant differences (*p* < 0.05).

**Figure 3:**
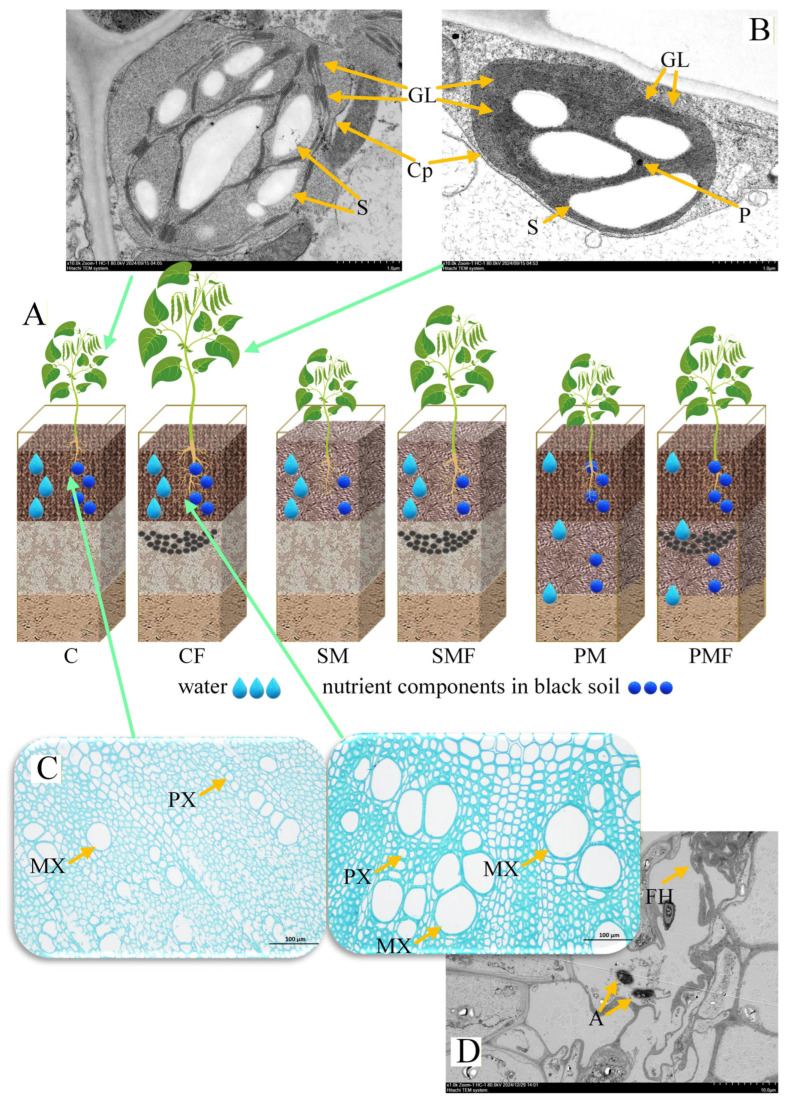
Histological and subcellular structures of soybean. (**A**) Schematic redistribution of water and nutrients in the plexiglass boxes. (**B**) Chloroplast ultrastructure of C and CF leaves. Cp, chloroplast; GL, granum lamella; S, starch granule; P, plastoglobuli; scale bar = 1 µm. (**C**), Root cross-sections of C and CF. PX, protoxylem; MX, metaxylem; scale bar = 100 µm. (**D**), TEM micrograph of CF roots. A, arbuscule; FH, fungal hyphae; scale bar = 10 µm.

At 10 days after sowing, no seeds had emerged in either PM or PMF; 4 seedlings were transplanted into each box. Figure 1C illustrates growth on day 35. Harvest on 20 September revealed roots extending to 50 cm. Figure 2 quantifies the impact of deep placement fertilization on soybean performance. The number of nodes did not differ among treatments. Throughout the experiment, CF exhibited a significantly greater plant height, stem thickness, number of grains, and yield per plant than C. In the first 35 days, SMF and SM were indistinguishable, but at harvest SMF surpassed SM in the number of grains and yield. PMF never differed from PM, presumably because water and nutrients were redistributed within the box (Figure 3A).

### 3.2. Effect of Fertilization on Soybean Tissue and Subcellular Structure

No overt histological differences were detected between CF and C stems or leaves (Supplementary Figure S1A); however, CF exhibited a significantly wider metaxylem (Figure 3C). The chloroplast ultrastructure revealed an increased number of grana lamellae in CF compared to C (Figure 3B), whereas TEM of stem tissues showed no difference (Supplementary Figure S1B). Arbuscular mycorrhizal fungi (AMF) were present in root cortical cells of CF (Figure 3D).

### 3.3. Effects of Fertilization on the Soybean Transcriptome, Proteome, and Metabolome

**Figure 4:**
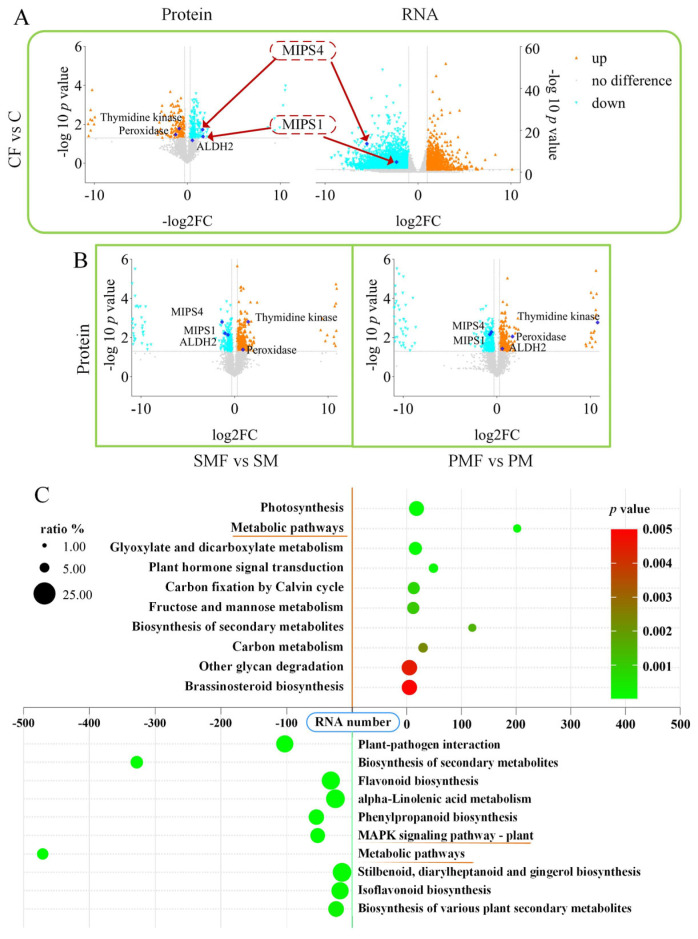
Transcriptomic and proteomic alterations in soybean. (**A**) Volcano plots of transcriptome and proteome data for CF vs. C. (**B**) Volcano plots of proteome data for SMF vs. SM and PMF vs. PM. (**C**) KEGG enrichment in CF vs. C. Positive RNA numbers indicate upregulation, negative numbers indicate downregulation.

Figure 4A shows that MIPS1 and MIPS4 were downregulated in CF vs. C at both transcript and protein levels; the same proteins were also repressed in SMF vs. SM and PMF vs. PM (Figure 4B). In all three contrasts, peroxidase (PX/POD) and thymidine kinases (TKs) were consistently upregulated. ALDH2 (aldehyde dehydrogenase 2) was downregulated in SMF vs. SM but upregulated in PMF vs. PM. No differential expression of these genes was detected at the RNA level in either SMF vs. SM or PMF vs. PM (Supplementary Figure S2A).

Transcriptomic KEGG enrichment revealed upregulated genes converging on “metabolic pathways” and downregulated genes converging on the “MAPK (mitogen-activated protein kinase) signaling pathway” in all three contrasts (Figure 4C and Figure S2B,C).

**Figure 5:**
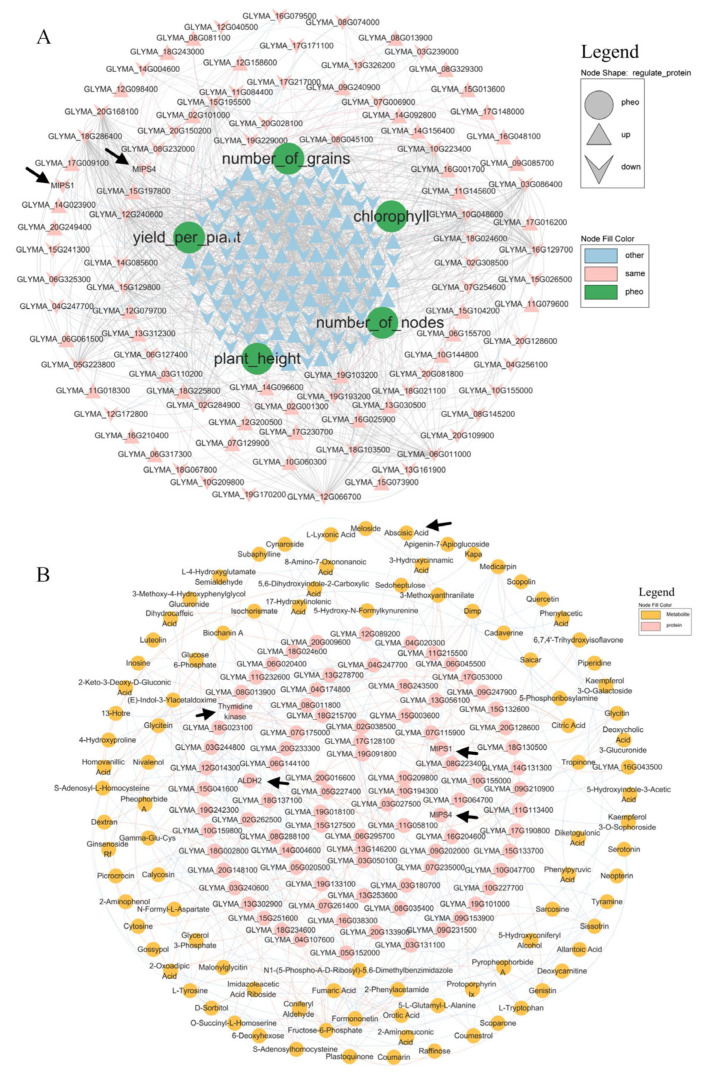
Integration of transcriptome, proteome, and metabolome data with the phenotype. (**A**) mRNA–protein–phenotype network. Pink nodes: consistent transcript–protein trend; light-blue: others; green: phenotypic traits. Grey edges: protein–protein; light-blue: negative protein–phenotype; light-red: positive; line thickness denotes correlation strength. (**B**) Protein–metabolite correlations. Light-blue edges: negative; light-red: positive; thickness reflects correlation strength.

**Figure 6:**
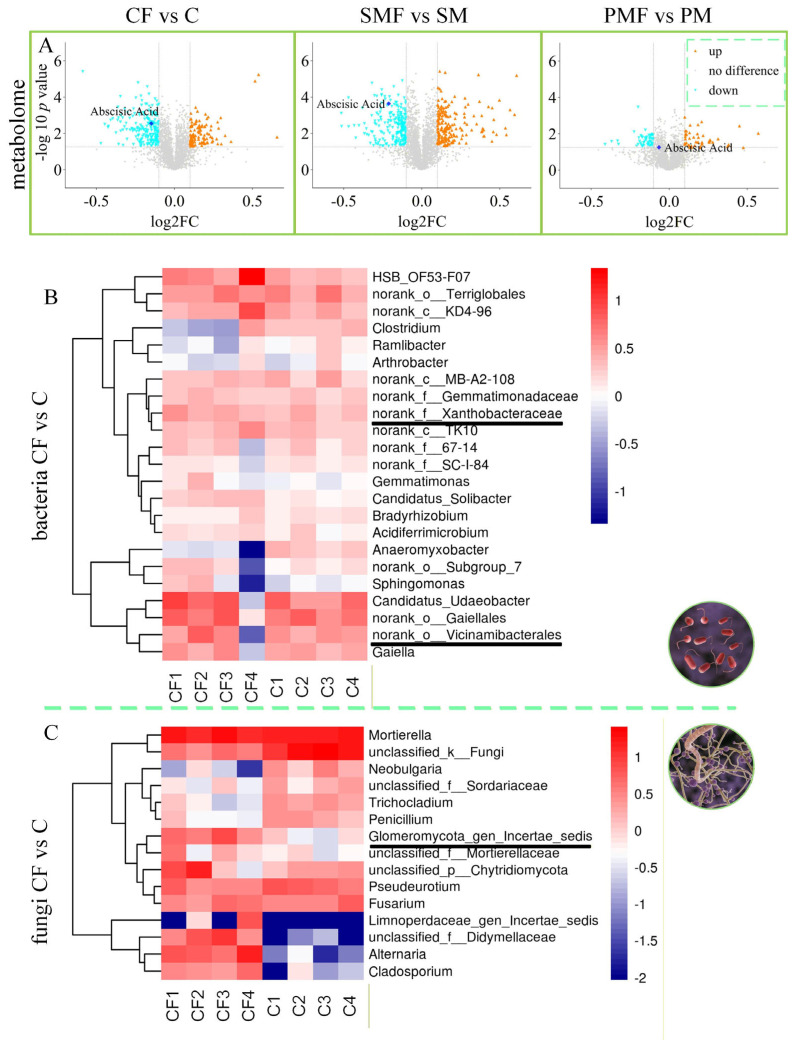
Shifts in soybean metabolites and rhizosphere microbiota. (**A**) Volcano plots of metabolites for the three pairwise comparisons. (**B**) Heat map of rhizosphere bacterial contrasts. (**C**) Heat map of rhizosphere fungal contrasts.

mRNA–protein–phenotype correlations (Figure 5A) revealed that MIPS1/MIPS4 (myo-inositol 1-phosphate synthase) was negatively associated with the yield per plant. DEM mapping (Figure 6A) showed that abscisic acid (ABA) was downregulated in both CF vs. C and SMF vs. SM. Protein–metabolite integration (Figure 5B) indicated no direct correlation between the five target proteins and ABA.

### 3.4. Rhizosphere Microbiome of Soybean

**Figure 7:**
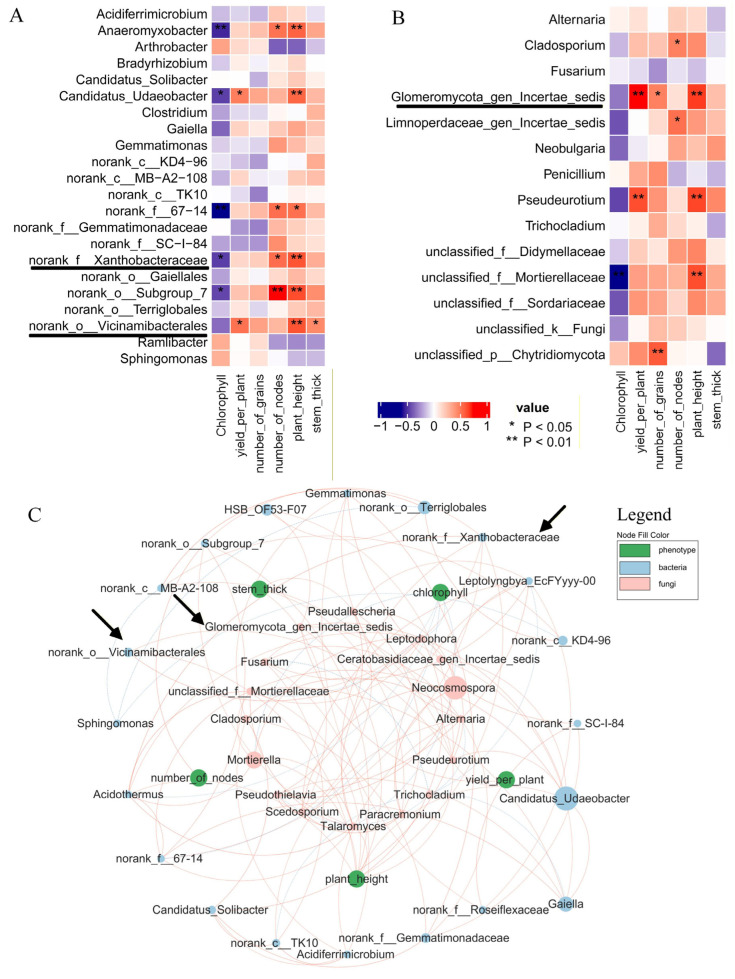
Correlation between soybean metabolites and rhizosphere microbiota. (**A**) Correlation between soil bacteria and phenotypic traits. (**B**) Correlation between soil fungi and phenotypic traits. (**C**) Integrated correlations among bacteria, fungi, and phenotypes. Pink nodes: fungi; light-blue nodes: bacteria; green nodes: phenotypes. Light-blue edges: negative correlations; red edges: positive correlations. Node area reflects relative abundance.

Rhizosphere soil bacteria (Figure 6B) showed elevated abundances of *Xanthobacteraceae* and *Vicinamibacterales* in CF vs. C, with log_2_FC = 0.1549 and 0.3275, respectively. Fungal data (Figure 6C) revealed a log_2_FC of 2.7257, showing an increase in *Glomeromycota_gen_certaes_dedis* in the same comparison. SMF vs. SM and PMF vs. PM obtained similar results (Supplementary Figures S3 and S4). Based on previous studies about AMF genera [33,34,35], *Glomeromycota_gen_certaes_dedis*, identified in this study was an unnamed AMF, hereinafter referred to as *Glomeromycota*. Figure 7A,B demonstrates that all three taxa were positively correlated with plant height (*p* < 0.01). Figure 7C indicates no direct intercorrelation among *Vicinamibacterales*, *Xanthobacteraceae*, and *Glomeromycota*.

### 3.5. Effect of the Proposed Mode of Deep Placement Fertilization on Soybean

**Figure 8:**
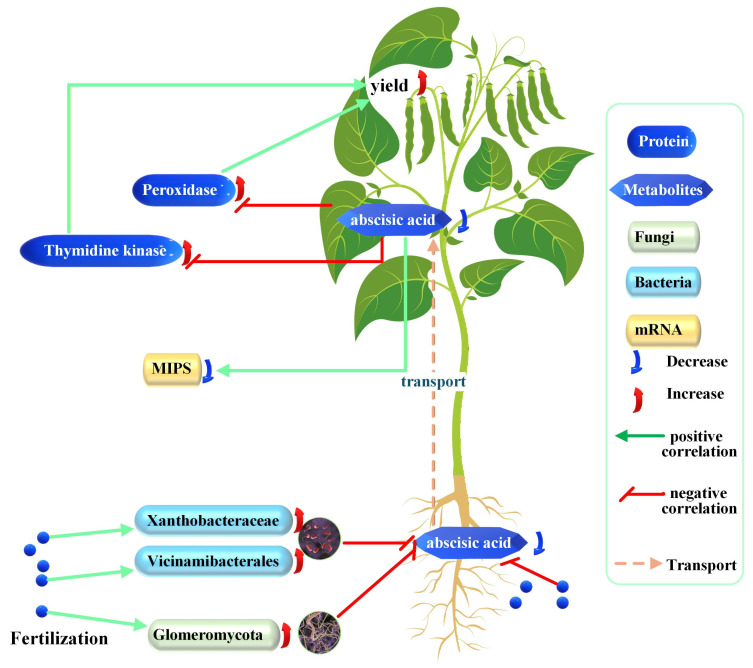
Proposed model of the effects of fertilizer on soybean development. Each correlation depicted is discussed and theoretically supported in the text; increases and decreases are experimental observations of this study.

Integrating this study data with published reports, the study proposes a model in which deep-placed fertilizer reshapes the soybean rhizobiont, suppressing root ABA synthesis and subsequent ABA translocation to leaves. Reduced foliar ABA elevates TK and POD activities, increasing yield (Figure 8).

## 4. Discussion

Albic black soil is an inherently low-fertility pedon [11,13,14,15] whose available boron is immobilized in stagnic fluvisol clay complexes that reduce the yield of *Beta vulgaris* L. [17]. Reclamation must progressively increase fertility because the surface black horizon is often <20 cm [16]; conventional 20 cm rotary tillage dilutes it with the albic layer, reducing the yield. Previous amelioration relied on surface fertilizer or straw incorporation [13], ignoring the rapid leaching of nutrients into downstream water. This study offers a deep placement technology that positions fertilizer within the impermeable albic layer, minimizing hydrologic loss (Figure 1A).

Tillage and fertilization reassemble soil microbiomes; plant acquisition of organic nutrients is tightly coupled to highly diverse microbial consortia [36,37]. Repeated fertilization and cultivation increase the soil organic carbon, and its accrual drives the formation of organo-mineral aggregates [11]. The root system of plants can also improve soil permeability. After planting *Caragana korshinskii*, the root system of plants showed a significant positive correlation with soil macropores and soil mineral particles [38]. Plant–soil–microbiome interactions remain poorly understood [39], prompting the rhizobiont concept, which integrates plant–root–rhizosphere–mycorrhizosphere–bulk soil and their resident microbes [10]. In the rhizobiont, cytokinins operate as shared signals; these phytohormones are synthesized by plants, bacteria, fungi, insects, and earthworms, promoting plant growth and soil health [40]. The commonly used cultivation method in Heilongjiang Province is rotary tillage at a depth of 20 cm, and the bottom fertilizer is applied 5 cm below the seed side during ridge sowing [41]. This depth of fertilization allows seeds to quickly receive fertilizer support after germination, resulting in higher soybean yields than deep fertilization. However, this method results in a significant loss of fertilizer due to the migration of soil moisture in white soil. Deep fertilization not only changes soil fertility but also alters the physical and chemical properties of soil mixed with white matter layer. This is a very complex situation that was not explored in detail in this study.

Deep placement fertilization first restructures the soil microbiome, in which biodiversity is the primary driver of plant growth [42,43]. *Vicinamibacterales* actively mediate soil nitrogen metabolism, promoting nitrogen retention [44], and its abundance in the soybean rhizosphere is positively correlated with the total soil nitrogen [45,46]. *Xanthobacteraceae* is a typical rhizobacterial participant in soil nitrogen deposition and cycling [47]. Under cadmium stress, it cooperates with *Bradyrhizobium* to sustain nodule nitrogen fixation [48], and its abundance in the olive rhizosphere positively correlates to plant growth [49]. Consistently, the fertilization regime used in this study increased the relative abundances of *Vicinamibacterales* and *Xanthobacteraceae* (Figure 6B), both of which were positively correlated with plant height and stem thickness (Figure 7A).

*Glomeromycota* are beneficial microorganisms in soil that promote soybean growth [50]; this phylum of *Glomeromycota* comprises mostly AMF genus [33,34,35]. AMF belonging to several families within *Glomeromycota* effectively enhance soybean resistance to *Heterodera glycines* [51] and, through reciprocal nutrient exchange, stimulate rhizobia–legume symbiosis [52]. Consistent with these reports, fertilization significantly increased the relative abundance of *Glomeromycota* (Figure 6C), which was positively correlated with plant height and yield (Figure 7B). Moreover, AMF structures were observed in fertilized roots (Figure 3D). Increasing AMF in soil can reduce the relative concentration of ABA in the root tissue of Liquorice (*Glycyrhiza uralensis*) [53]. Of course, some studies suggest that endogenous ABA regulates AMF on rooting of tea plants (*Camellia sinensis* L.) cuttings [54]. This study found that *Glomeromycota* (AMF) increased in the rhizosphere soil, which further confirmed a decrease in root ABA content.

Fertilizer acts directly or via microbe-mediated signals on soybean roots, causing anatomical changes (Figure 3C). Metabolomic profiling detected a fertilization-induced decrease in the foliar content of ABA (Figure 6A), a sesquiterpene hormone that negatively regulates soybean growth [55]. ABA synthesized in roots is transported to leaves through xylem sap and triggers drought-responsive pathways that suppress expansion [56]; ABA receptor knockouts are taller than the wild type [57], explaining the enhanced metaxylem development observed in fertilized roots. Studies have shown that ABA is generated in the roots, especially when ABA is secreted vigorously at the end of the roots, regulating root development [58,59,60]. Potatoes lacking ABA cannot undergo normal tuber development [61]. Then, ABA is transported from the roots to the leaves, and currently three ABA output proteins have been identified: Arabidopsis ATB-Binding Cassette G25 (AtABCG25), AtABCG31 and Detoxification Efflux Carrier 50 (AtDTX50) [62]. The indirect evaluation of ABA synthesis in roots by measuring ABA concentration in leaves is an acceptable method in this study.

ABA suppresses xylem fiber differentiation in Arabidopsis hypocotyls [63], impedes sap transport through the xylem in *Medicago truncatula* to aphids under drought [64], and correlates negatively with the xylem vessel size in maize exposed to drought, salinity, or elevated CO_2_ [65]. The mechanism by which fertilization reduces the ABA content remains unresolved; in barley, excess nitrogen downregulates *HvMADS27*, inducing *HvBG1* and diminishing the root ABA content [66]. Thus, the study posits that ABA functions as the root-to-shoot signal mediating the fertilization response.

This study proposes a moisture–nutrient redistribution model by integrating the six treatments (Figure 3A). CF combines optimal water and fertilizer, resulting in the greatest biomass; SMF is nutrient limited. PM is so water limited that seedlings failed and required transplanting. ABA conveys the root-level moisture–nutrient status to the shoot, and its concentration decreases under fertilization. Proteomics revealed that MIPS1 and MIPS4 were reduced in CF leaves (Figure 6A). ABA induces *MIPS1* expression in sweet potato [67]. Legume *MIPS* genes carry ABA-responsive elements positively linked to their expression [68], and exogenous ABA upregulates *MIPS1/2* in chickpea (*Cicer arietinum* L.) seedlings [69]. In *Spirodela polyrrhiza* [70], MIPS1 also promotes soybean germination. Wild-type lines significantly outperform *MIPS1*-null mutants [71], and recent mutant panels confirm that loss-of-function alleles reduce emergence rates [72], corroborating the moisture–nutrient redistribution model generated in this study.

Soybean leaf proteomics revealed elevated relative abundances of TKs and POD following fertilization (Figure 4A,B). TK catalyzes the salvage of thymidine to thymidine 5′-monophosphate, supplying nucleotides for DNA synthesis and repair throughout plant development [73]; this salvage activity promotes growth at all developmental stages [73,74]. POD has a growth-promoting effect [75] and is positively correlated with the number of soybean nodes [76]. The *Sinorhizobium* HH103 NopT type III effector modulates POD levels, enhancing soybean growth [76].

Upregulated genes were enriched in “metabolic pathways”, whereas downregulated genes converged on “MAPK signaling pathway” (Figure 4C). The mitogen-activated protein kinase cascade is a conserved module that drives plant growth, development, and stress responses [77]. MKK4 (mitogen-activated protein kinase 4) operates upstream of the MAPK cascade and positively regulates growth [78]; under stress, MKK4 is transcriptionally activated, enhancing soybean tolerance [79,80,81].

Water deficit in PM and PMF triggered a drought-response network in PMF that included upregulation of ALDH2. Aldehyde dehydrogenases (ALDHs) utilize NAD+/NADP+ to convert aromatic or aliphatic aldehydes into less toxic carboxylic acids, mitigating aldehyde injury [82]. Transcriptomic surveys have shown *ALDH* induction under drought in soybean and mustard [83,84], and similar increases have been reported for *Arabidopsis thaliana* and garlic subjected to water shortage [85,86].

Integrating the four omics layers reveals a pattern consistent with published reports. After soybean mosaic virus infection, ABA, POD, and the MAPK signaling pathway exhibit coordinated changes [87]. Similarly, sugar beet seedlings exposed to saline–alkali stress show an increased POD content and decreased ABA content following microbial fertilizer application [88]. Adding AMF to the soil increased Eggplant Peroxidase, which can better alleviate Root Rot Stress and increase yield [89]. AMF can enhance the drought resistance of maize, increase the content of peroxidase in leaves, and inhibit ABA signal pathways in roots [90]. After inoculation with AMF, lupine increased the content and yield of Peroxidase, and effectively inhibited Rhizoctonia root rot [91].

### Limitation

The causal physiological explanations remain unclear, and no hypothesis-driven work has been carried out, so that the manuscript remains descriptive. In addition, in the setting of the control experiment in this study, the broad application of fertilization in farmland was not considered, so the reference for extending the experimental results to medium range planting experiments has certain limitations.

## 5. Conclusions

The central challenge of feeding humanity is securing maximal grain yield on inherently infertile land, such as albic black soil. Here, deep placement fertilization increased the soybean yield by restructuring the rhizobiont, suppressing root ABA synthesis, and increasing foliar TK and POD activities. Among the fertilizer treatments, CF attained the best growth, and SMF suffered from nutrient shortage. SMF and PMF lost water faster than CF. PM lost water most rapidly, failed to support germination, and required seedling transplant. PM upregulated drought-protective protein ALDH2. These findings provide a mechanistic basis for small-scale trials of deep placement machinery. The albic horizon must be preserved to retain moisture. A 25 cm placement depth advances growth. Future work should quantify fertilizer residence in soil and verify that downstream eutrophication has been mitigated.

## Figures and Tables

**Table 1:** Experimental treatment design.

	Groups	Treatment	Fertilization *	Simulated Soil Profiles
1	Control (C)	Top: black soil 20 cm, middle: albic horizon 20 cm, bottom: illuvial horizon 10 cm	No	Simulation of albic soil capped by ≥20 cm black topsoil.
2	Control and fertilizer (CF)	Top: black soil 20 cm, middle: albic horizon 20 cm, bottom: illuvial horizon 10 cm	25 cm	
3	Surface soil mixing (SM)	Top: mixed layer (black soil + albic 1:1) 20 cm, middle: albic horizon 20 cm, bottom: illuvial horizon 10 cm	No	Simulation of albic soil capped by ≤10 cm black topsoil.
4	Surface soil mixing and fertilizer (SMF)	Top: mixed layer (black soil + albic 1:1) 20 cm, middle: albic horizon 20 cm, bottom: illuvial horizon 10 cm	25 cm	
5	Plow pan soil mixing (PM)	Top: black soil 20 cm, middle: mixed layer (black soil + albic 1:1) 20 cm, bottom: illuvial horizon 10 cm	No	Simulation of sub-soil tillage (20–40 cm) with surface horizon left undisturbed.
6	Plow pan soil mixing and fertilizer (PMF)	Top: black soil 20 cm, middle: mixed layer (black soil + albic 1:1) 20 cm, bottom: illuvial horizon 10 cm	25 cm	

* Fertilizer supply per fertilized box: urea, 3 g; diammonium phosphate, 7.5 g; potassium sulfate, 3.8 g. Boxes were placed outdoors with natural rainfall as the main source of water replenishment; after sowing, 200 mL of tap water was applied to each box with a beaker. During rain-free periods, irrigation (200 mL per box) was triggered when the surface soil (2 cm) in the control group became dry.

## Data Availability

All relevant data are contained within the manuscript.

## Supplementary Materials

The following supporting information can be downloaded at https://www.mdpi.com/article/10.3390/plants15030424/s1.
