# Supplementary figures and images for: Effect of Deep Placement Fertilization on Soybean (Glycine max L.) Development in Albic Black Soil
Source: Plants (Basel). 2026 Jan 30;15(3):424. doi: 10.3390/plants15030424 (PMC12900028; doi:10.3390/plants15030424)

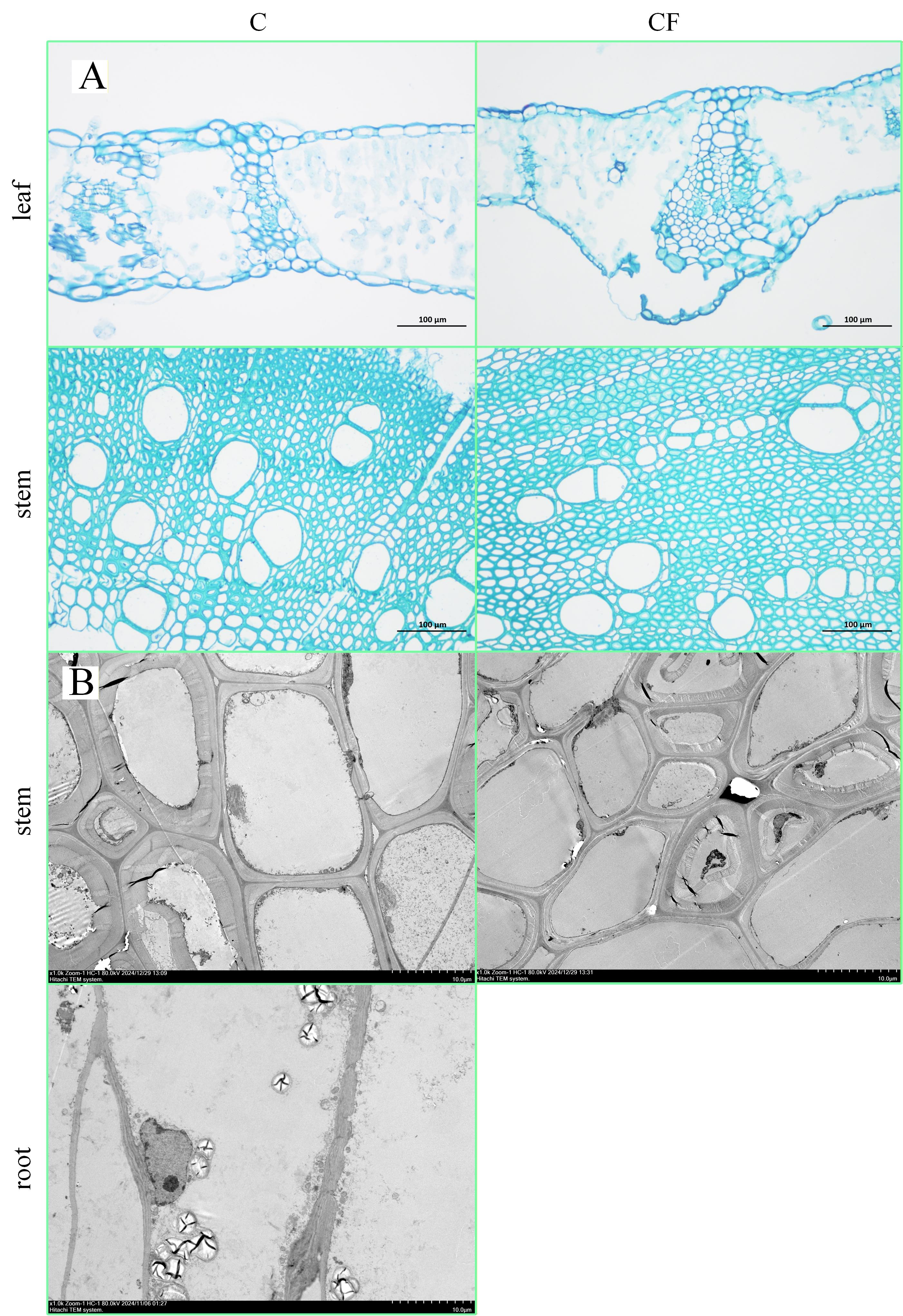

Supplement: Supplementary file 1 [file plants-15-00424-s001.zip › Supplementary Figure S1.jpg]

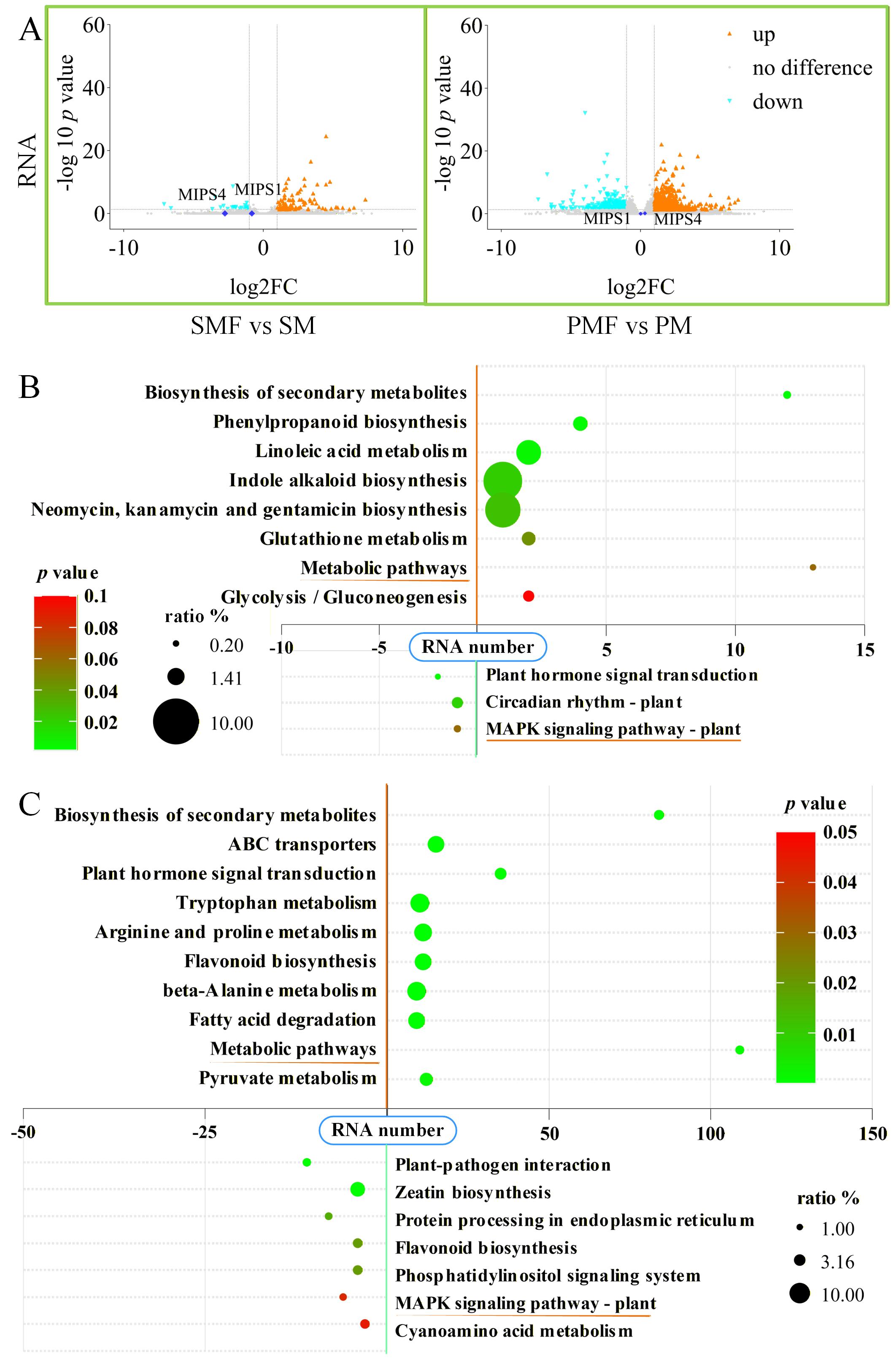

Supplement: Supplementary file 1 [file plants-15-00424-s001.zip › Supplementary Figure S2.jpg]

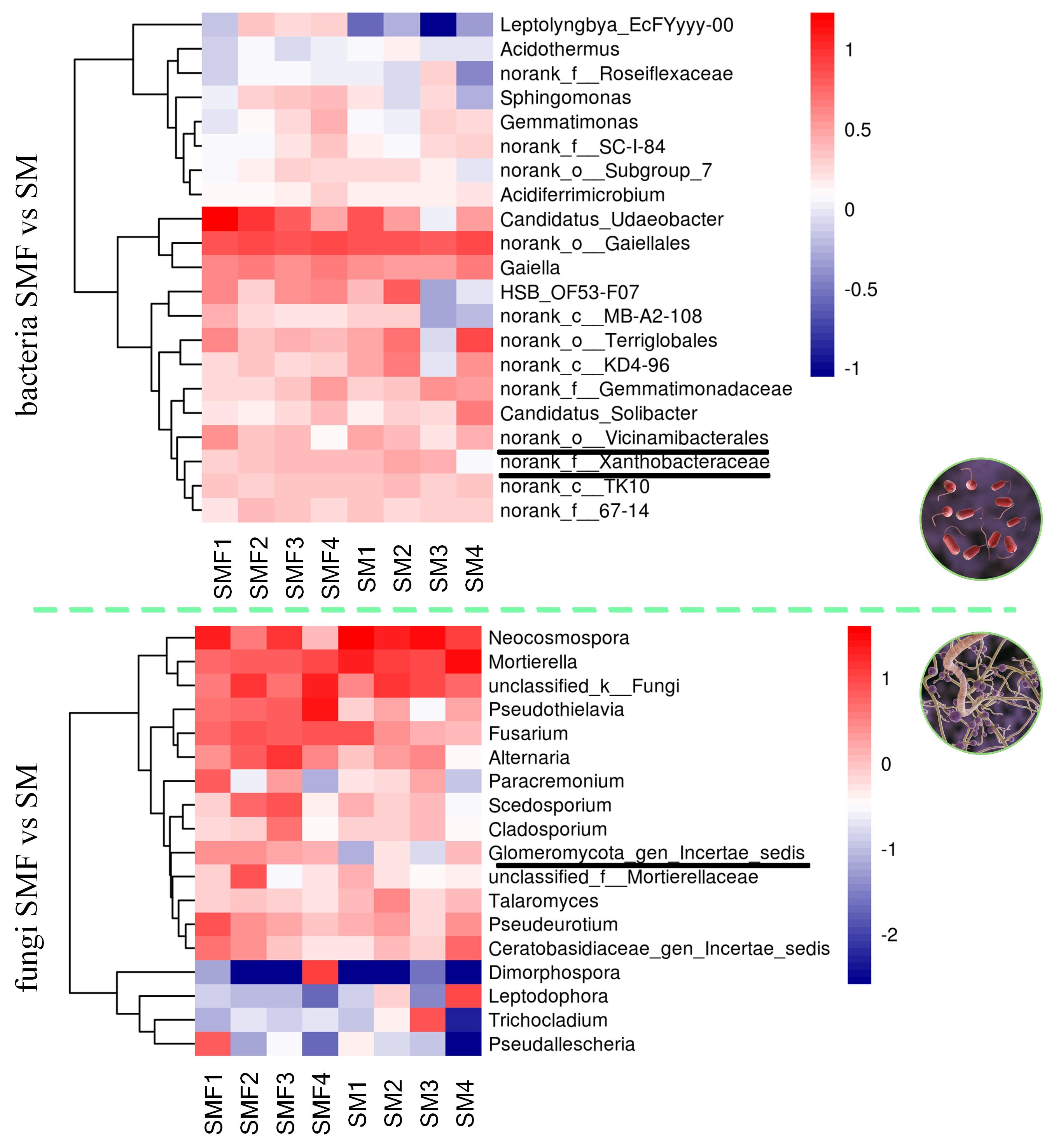

Supplement: Supplementary file 1 [file plants-15-00424-s001.zip › Supplementary Figure S3.jpg]

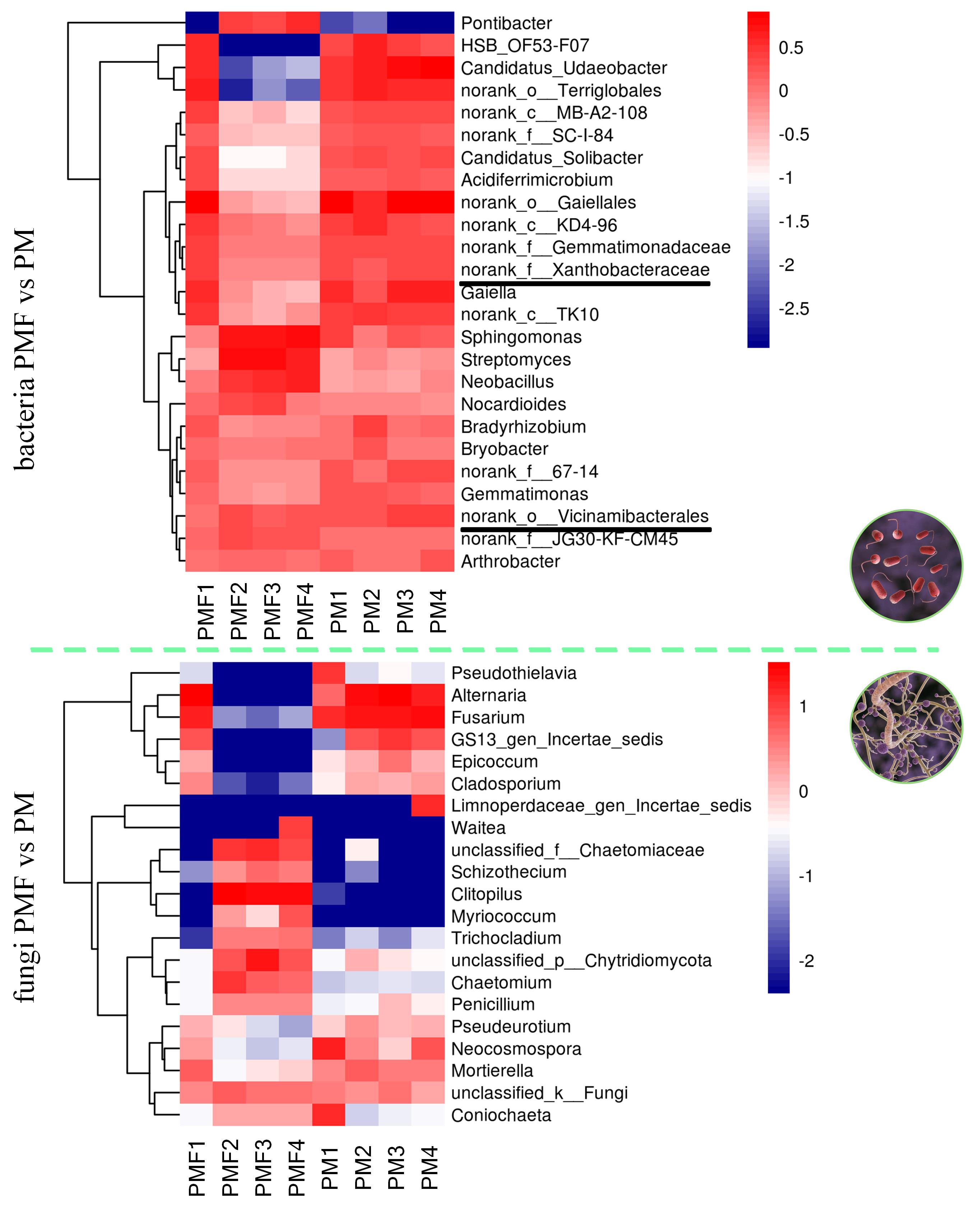

Supplement: Supplementary file 1 [file plants-15-00424-s001.zip › Supplementary Figure S4.jpg]
